# From Welfare to Warfare: The Arbitration of Host-Microbiota Interplay by the Type VI Secretion System

**DOI:** 10.3389/fcimb.2020.587948

**Published:** 2020-10-19

**Authors:** Thomas E. Wood, Ezra Aksoy, Abderrahman Hachani

**Affiliations:** ^1^Division of Infectious Diseases, Department of Medicine, Massachusetts General Hospital, Boston, MA, United States; ^2^Department of Microbiology, Harvard Medical School, Boston, MA, United States; ^3^Centre for Biochemical Pharmacology, William Harvey Research Institute, Queen Mary University of London, London, United Kingdom; ^4^Department of Microbiology and Immunology, University of Melbourne at the Peter Doherty Institute for Infection and Immunity, Melbourne, VIC, Australia

**Keywords:** gut microbiome, type six secretion system, commensal, symbiosis, dysbiosis, mucosal immunity, tolerance, MAMPs

## Abstract

The health of mammals depends on a complex interplay with their microbial ecosystems. Compartments exposed to external environments such as the mucosal surfaces of the gastrointestinal tract accommodate the gut microbiota, composed by a wide range of bacteria. The gut microbiome confers benefits to the host, including expansion of metabolic potential and the development of an immune system that can robustly protect from external and internal insults. The cooperation between gut microbiome and host is enabled in part by the formation of partitioned niches that harbor diverse bacterial phyla. Bacterial secretion systems are commonly employed to manipulate the composition of these local environments. Here, we explore the roles of the bacterial type VI secretion system (T6SS), present in ~25% of gram-negative bacteria, including many symbionts, in the establishment and perturbation of bacterial commensalism, and symbiosis in host mucosal sites. This versatile apparatus drives bacterial competition, although in some cases can also interfere directly with host cells and facilitate nutrient acquisition. In addition, some bacterial pathogens cause disease when their T6SS leads to dysbiosis and subverts host immune responses in defined animal models. This review explores our knowledge of the T6SS in the context of the “host-microbiota-pathogen” triumvirate and examines contexts in which the importance of this secretion system may be underappreciated.

## Introduction

The gut tissue is composed of hundreds of millions of cells whilst providing a home for a microbiota containing trillions of bacteria (Sender et al., 2016). The association of the microbiota with our tissues is central for homeostatic and developmental mechanisms and thus governs many aspects of human health (Belkaid and Harrison, 2017). Due to this relationship, mammals in general may be considered as holobionts from an ecological perspective, in which the microbiota assists host metabolism and acts as an environmental training system for the associated tissues (Bäckhed et al., 2005; Al Nabhani et al., 2019; Tsolis and Bäumler, 2020). Microorganisms associate with the skin and mucosal surfaces such as the oral-nasal and vaginal cavities, respiratory and gastrointestinal tracts; with the gut microbiota constituting the best characterized community. We note that although we focus on the gut microbial ecosystem, the concepts may apply to all mucosal surfaces and potentially to other complex symbiotic communities.

The composition and community structure of the gut microbiota is complex and heterogenous. The distribution of microbial species within the large intestine is to be accounted with the diversity of residing immune cells, together forming a biodynamic ecosystem (Human Microbiome Project Consortium, 2012; James et al., 2020). Indeed, bacterial communities and immune cell populations exhibit great diversity in a niche-dependent fashion, with the latter displaying a wide range of transcriptional profiles within T and B cells of the adaptive immune system. The niches of gut commensals are determined by their metabolic activities and ability to stably associate with their local tissue environment (Lee et al., 2013; Ost and Round, 2018; Vonaesch et al., 2018). For example, some of the *Bacteroides* species are present in the intestinal lumen while others tightly associate with the mucus layering the epithelial surface of colonic crypts (Johansson et al., 2011). Yet, niche residency is not solely determined through dialogue with the host and critically depends on interactions with other microbes sharing nutritional niches (García-Bayona and Comstock, 2018). Here, bacteria vie for dominance, deploying a range of antibacterial toxins, some of which are delivered *via* membrane-embedded secretion systems.

The T6SS is prevalent in gram-negative bacteria, particularly in the phyla Proteobacteria and Bacteroidetes (Bingle et al., 2008; Russell et al., 2014b). This secretion apparatus is evolutionarily related to the bacteriophage tail, wherein contraction of a sheath propels a spiked-tube structure out of the bacterial cell, piercing the cell membrane of their targets to inject effector proteins (Pukatzki et al., 2007; Coulthurst, 2019). The cytoplasmic T6SS sheath, composed of a polymeric helix of TssB-TssC binds to a baseplate-like multi-protein platform, which itself associates with an envelope-spanning membrane complex of TssJ, TssL, and TssM (Durand et al., 2015; Nazarov et al., 2017). Phylogenetic analysis of TssC proteins found that type VI secretion systems cluster into three main groups, where subtypes I and II are proteobacterial, while subtype III is restricted to Bacteroidetes (Russell et al., 2014b). The inner tube is a stack of hexameric Hcp rings capped with a spike complex of a VgrG trimer, further sharpened with a PAAR protein tip; designed for effector and toxin delivery (Leiman et al., 2009; Shneider et al., 2013). T6SSs can directly target both prokaryotic and eukaryotic cells, as well as delivering effector proteins into the extracellular milieu in a contact-independent manner (Pukatzki et al., 2006; Hood et al., 2010; Si et al., 2017b). These effectors display a vast range of activities, including hydrolysis of peptidoglycan of peptidoglycan, nucleic acids, nucleotides, proteins, and lipids; membrane pore formation and metal ion binding, thus conferring a competition advantage to the T6SS-wielding bacterium and promoting its survival (Russell et al., 2014a; Wang et al., 2015; Ahmad et al., 2019). This review examines the relationship between the type VI secretion system and the microbiome in the context of both symbiosis and dysbiosis.

## The T6SS Contributes to the Fitness of the Microbiota

The majority of the mammalian microbiome is acquired at birth, with the prevailing species seeded from the mother during delivery and influenced by breastfeeding and environmental exposure (Round et al., 2010). During the first year of life, the composition of the gut microbiome is highly dynamic, in part due to the weaning process, before stabilizing, and remaining consistent through adulthood (Faith et al., 2013; Verster et al., 2017; Al Nabhani et al., 2019). The major constituents of the gut community belong to the phyla Bacteroidetes, Firmicutes, Actinobacteria, and Proteobacteria, with members of the *Bacteroides* genus dominating the large intestine (Human Microbiome Project Consortium, 2012). Subtype III of T6SS (hereafter referred as T6SS^iii^) is restricted to the Bacteroidetes phylum and has been shown to deliver antibacterial effectors resulting in microbial antagonism (Russell et al., 2014b).

Bioinformatic analyses of T6SS loci within the order Bacteroidales has classified them into three distinct “genetic architectures,” designated GA1–3 (Coyne et al., 2016). GA1 and GA2 are found on integrative conjugative elements. Genomic analysis of the co-resident *Bacteroides* spp. isolated from human gut provided evidence of transfer of these elements between species *in situ*, implying that T6SS loci are under positive selection in the microbiome (Coyne et al., 2014). GA3 T6SSs are confined to *Bacteroides fragilis*, an obligate anaerobe, while GA1 and GA2 loci are more widespread within the phylum (Coyne et al., 2016). GA1–3 display distinct repertoires of effector-immunity pairs, possibly driving the incompatibility of these T6SSs within a single niche of an individual (Coyne and Comstock, 2019). One strain of *B. fragilis* tends to dominate the microbiota of an individual due to strain exclusion as the composition of the community stabilizes (Kostic et al., 2015; Yassour et al., 2016; Verster et al., 2017). Indeed, metagenomic analyses revealed that the abundance of GA3 T6SS loci is higher in infants, suggesting that competition between *B. fragilis* strains leads to stability of the microbial community in adulthood (Coyte et al., 2015; Verster et al., 2017). These observations should also be considered in light of the weaning process, wherein dietary changes lead to the influx of new bacterial competitors and dietary metabolites required for the host immune ontogeny (Al Nabhani et al., 2019). Co-existence of strains with different T6SS^iii^ “genetic architectures” does arise but solely when bacterial species with overlapping nutritional niches become spatially segregated in the presence of a dense and diverse microbiota (Zitomersky et al., 2011; Hecht et al., 2016).

The use of gnotobiotic mouse models provided the empirical evidence supporting the roles of the T6SS in Bacteroidetes as ecological determinants, wherein T6SS expression and activity have been directly detected *in vivo* (Russell et al., 2014b; Chatzidaki-Livanis et al., 2016). *In vivo* competition assays have demonstrated that *B. fragilis* employs the T6SS to displace competitors from their niche in a contact-dependent manner, with several effector proteins supporting this elimination (Table 1) (Chatzidaki-Livanis et al., 2016; Hecht et al., 2016; Wexler et al., 2016; Ross et al., 2019). Furthermore, *in vitro* competition assays have found that T6SS-mediated antagonism of *Bacteroides* spp. targeted a narrow range of species, with most prey strains resistant to intoxication (Chatzidaki-Livanis et al., 2016; Wexler et al., 2016). Thus, the susceptibility to T6SS-dependent antagonism depends as much on the belligerent's identity as on the population distribution across topological niches.

**Table 1:** T6SS^iii^ effectors of human symbionts.

**Commensal bacterium**	**Antibacterial T6SS**	**Effector locus**	**Immunity protein(s)**	**Immunity locus**	**References**
	**effector**	**tag**	**tag(s)**		
*Bacteroides dorei* DSM 17855	“GA2_e14”	BACDOR_RS22955	“GA2_i14”	BACDOR_RS17020	Ross et al., 2019
*Bacteroides fragilis* 638R	Bfe1	BF638R_1988	Bfi1	BF638R_1987	Chatzidaki-Livanis et al., 2016
*Bacteroides fragilis* 638R	Bfe2	BF638R_1979	Bfi2	BF638R_1978	Chatzidaki-Livanis et al., 2016
*Bacteroides fragilis* 638R	–	–	Orphan Bti1 (“GA3_i6”)	BF638R_2042	Ross et al., 2019
*Bacteroides fragilis* 638R	–	–	Orphan Bti2a,b (“GA3_i7ab”)	BF638R_2053-4	Ross et al., 2019
*Bacteroides fragilis* 638R	–	–	Orphan “GA2_i11”	BF638R_1388	Ross et al., 2019
*Bacteroides fragilis* CL03T00C23	“GA2_e2”	HMPREF1079_RS08215	“GA2_i2”	HMPREF1079_RS08220	Ross et al., 2019
*Bacteroides fragilis* NCTC 9343	Bte1 (“GA3_e6”)	BF9343_1937	Bti1 (“GA3_i6”)	BF9343_1936	Wexler et al., 2016
*Bacteroides fragilis* NCTC 9343	Bte2	BF9343_1928	Bti2a,b (“GA3_i7ab”)	BF9343_1927-6	Hecht et al., 2016; Wexler et al., 2016
*Bacteroides fragilis* NCTC 9343	–	–	Orphan “GA1_i5”	BF9343_1657	Ross et al., 2019
*Bacteroides fragilis* YCH46	“GA1_e5”	BF2850	“GA1_i5”	BF2851	Ross et al., 2019

Horizontal gene transfer facilitates the evolution of bacterial species in polymicrobial environments by enabling the positive selection of genes conferring a competitive advantage, a phenomenon also observed for T6SS loci (Unterweger et al., 2014). The existence of “orphan” T6SS immunity genes (conferring resistance to deleterious T6SS effector proteins; bearing no connection to the host immune system) in the absence of cognate effector genes was discovered in *Vibrio cholerae* isolates, leading to the hypothesis that their acquisition would subsequently protect the bearer against T6SS attacks from non-kin opponents (Kirchberger et al., 2017). The functionality of these orphan immunity genes was elegantly shown by Ross and colleagues in a recent study of members of the microbiome exhibiting extensive arrays called acquired interbacterial defense (AID) clusters (Ross et al., 2019). Here, many members of Bacteroidales were immune to T6SS antagonism by other species and may even possess immunity genes conferring resistance to anti-bacterial effectors associated to strategies beyond the T6SS (Zhang et al., 2012; Ross et al., 2019). However, immunity proteins are not the only way to mitigate the impact of antagonistic effectors. Several studies showed the inability of certain T6SS effectors to intoxicate prey cells lacking the cognate immunity proteins (Altindis et al., 2015; Ringel et al., 2017; Wood et al., 2019), and synergistic effector activities have also been described (LaCourse et al., 2018). Further protection strategies from T6SS-mediated killing, such as upregulation of envelope stress responses and production of extracellular polysaccharides, underscore the complexity of T6SS antagonism (Toska et al., 2018; Hersch et al., 2020).

T6SS-mediated bacterial antagonism targets specific competitors in the gut, helping to dictate niche occupancy. However, when considered in the broader ecological context of the microbiota and symbiosis with the host, the T6SS may also promote the symbiotic relationship with the host by enabling metabolic cooperation (Hooper et al., 2012; Vonaesch et al., 2018). Additionally, the presence of a stable microbiota provides resistance to dysbiosis and outcompetes invading microbial pathogens for nutrients. In terms of direct antibacterial warfare, the T6SS should be considered as a major armament of the microbiota in limiting infection (Kamada et al., 2013; Ducarmon et al., 2019). Indeed, mouse models have shown that the priority benefit of *B. fragilis* colonization may be protection against infection by enterotoxigenic *B. fragilis* strains, in a manner that depends on T6SS effector-immunity genotype (Hecht et al., 2016).

## Promotion of Immune Homeostasis by the Microbiota: A Potential Role for the T6SS?

**Figure 1:**
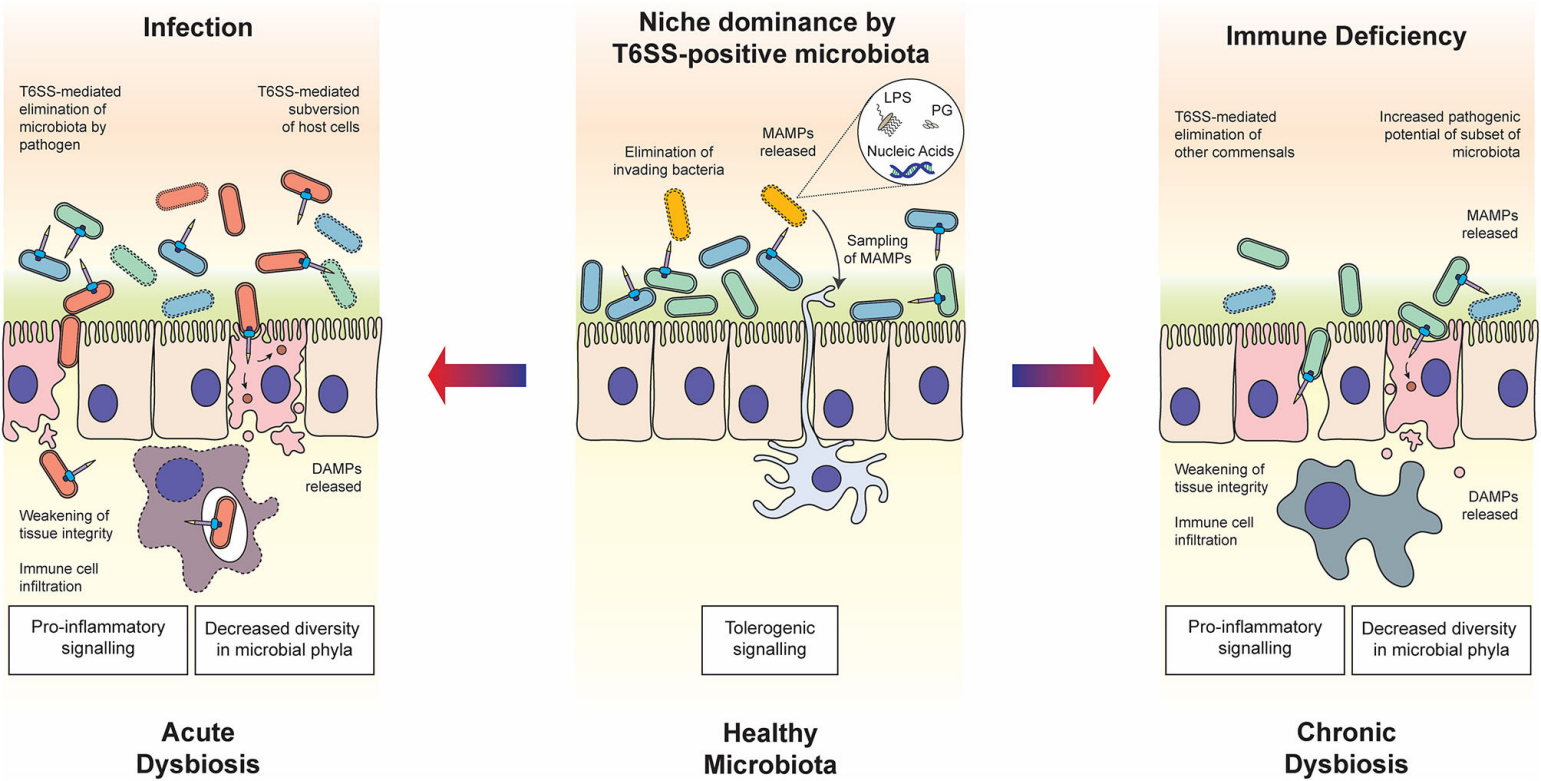
Roles of the T6SS in host-microbiota-pathogen interactions. In healthy steady state conditions (middle panel), commensal bacteria use the T6SS to establish and maintain their niche in the host. The release of MAMPs through T6SS warfare can contribute to the establishment of immune tolerance, enhancing the symbiotic relationship. In the case of host immune deficiency (right panel), for example due to a genetic polymorphism in the host, cross-talk with the microbiota is compromised and the balance within the microbial community may be disrupted, resulting in chronic dysbiosis. The T6SS is likely to play a role in the modulation of competing commensal populations and subsequent decrease in diversity of bacteria phyla, as well as potentially directly manipulating host cells. In the case of infection by pathogenic bacteria wielding a T6SS (left panel), commensal bacteria are eliminated through both direct delivery of antibacterial effectors and indirect mechanisms such as host manipulation and nutrient competition. The state of dysbiosis that follows is acute but may be resolved through elimination of the pathogen by the host immune system. In both states of dysbiosis, the T6SS may play a determining role in eliciting the release of DAMPs, which influences the host immune response.

The intestinal microbiota is also crucial for the development of our immune system, as its absence leads to low antibody titer, poor glycosylation of mucosal surfaces, overt T_H_2 responses and defective development of gut-associated lymphoid tissue in germ-free mice (Smith et al., 2007). The resident microbiota is proposed to train our immune system to actively tolerate the presence of distinct commensals whilst providing robust resistance against invading bacterial pathogens; presenting the intriguing teleological argument that commensal bacteria co-opt the host immune system to defend their niche (Round and Mazmanian, 2009). Evidence now strongly supports the idea of tolerogenic immune responses to commensal flora, rather than specifically ignoring these residents (Round et al., 2010). Tolerance is fostered through the detection of microbe-associated molecular patterns (MAMPs) and microbial metabolites, and extends beyond the local environment of the gut to promote appropriate systemic immune responses (Figure 1) (Clarke et al., 2010; Chu and Mazmanian, 2013). The detection of conserved MAMPs by pattern recognition receptors (PRRs) is one of the foundations of the innate immune system. Innate immune cells, particularly antigen-presenting dendritic cells (DCs) sense their environment in peripheral organs through continuous uptake and sampling of exogenously acquired antigens (Iwasaki and Medzhitov, 2015). Upon microbial encounter, the engagement of PRRs by MAMPs elicits an inflammatory genes program, enhances antigen processing and presentation processes in DCs; all critical for T cell mediated immune responses against pathogens (Medzhitov and Janeway, 1999; Takeuchi and Akira, 2010; Iwasaki and Medzhitov, 2015). MAMPs include lipopolysaccharide (LPS), peptidoglycan, lipoproteins and nucleic acids that trigger MAP kinase and NF-κB signaling leading to pro-inflammatory responses (Fitzgerald and Kagan, 2020). Yet, there is precedent for MAMPs to assist in the development of tolerogenic signals. Mucosal DCs interacting with commensal bacterial components directly or through indirect acquisition of secreted outer membrane vesicles (OMVs) prime host regulatory T cells (Tregs), a subset of T cells promoting tolerance to both food and microbial antigens, thus dampening immune responses to the resident bacterial communities.

The homeostasis of the host-microbiota axis is maintained by continuous immune system monitoring (Belkaid and Harrison, 2017). The best characterized example of immune modulation is the production of polysaccharide A (PSA) by *B. fragilis*, which signals *via* Toll-like receptor 2 (TLR2) on dendritic cells. This stimulates the differentiation of Tregs, producing an immunosuppressive environment through the secretion of the cytokine IL-10 (Mazmanian et al., 2005; O'Mahony et al., 2008; Round et al., 2011). In IL-10 deficient mice, commensal bacterium *Helicobacter hepaticus* exhibits colitogenic potential in the presence of gut microbiota, which has been reported to be suppressed by the T6SS of this ε-proteobacterium (Mazmanian et al., 2008; Chow and Mazmanian, 2010; Bartonickova et al., 2013; Jochum and Stecher, 2020). This highlights the interplay of tolerogenic signaling and the T6SS of resident members of the microbiota; however, the mechanistic details of this interaction are yet to be explored.

Tolerogenic immune signaling is also stimulated by commensal metabolites including the short chain fatty acids (SCFAs) acetate, propionate, and butyrate (Parada Venegas et al., 2019); intermediates of vitamin B2 and B9; and amino acid metabolism (Kjer-Nielsen et al., 2012; Venkatesh et al., 2014; Sasabe et al., 2016). Recent work has started to shed light on the numerous benefits that production of SCFAs by commensal bacteria confer to the host. One consequence is the upregulation of oxidative host metabolism by utilization of SCFAs as a carbon source, which bolsters the hypoxic microenvironment at the colonocyte surface, favoring the growth of obligate anaerobes (e.g., *Bacteroides* spp.) and limiting propagation of facultative aerobes, such as invasive pathogens like *Escherichia coli* (Litvak et al., 2018; Zhang et al., 2019). In addition, SCFAs act directly *via* immune cell receptors to modulate T cell subset expansion and macrophage polarization (Schulthess et al., 2019). These compounds commonly promote IL-10 production and suppress inflammation; however, they may also contribute toward effector T cell differentiation, depending on the overall local immunological context (Zhang et al., 2019). On the other hand, microbial metabolites in the intestine may stimulate virulence programs of invading bacteria, with several two-component signal transduction systems in T6SS-positive pathogens having been shown to respond to SCFAs and other metabolites produced by the microbiome (Lawhon et al., 2002; Gonzalez-Chavez et al., 2010; Kohli et al., 2018; Goodman et al., 2020). It is likely that T6SS-mediated turbulent population dynamics occurring during the microbiome development results in variation in the levels of these metabolites. Indeed, bacteria activate diverse antimicrobial programs upon non-kin recognition or danger sensing, including an as-yet uncharacterized diffusible signal from lysed *Pseudomonas aeruginosa* bacteria that heightens the antibacterial T6SS activity in kin (LeRoux et al., 2015). This antibacterial warfare would further alter levels of microbial products in the local milieu, tipping the ecological balance toward dysbiosis. Moreover, one could hypothesize that bacterial products resulting from the aftermath of T6SS-mediated bacterial antagonism may provide the ligands supporting the development of tolerogenic immune responses. Several lines of evidence from various models lend support to this hypothesis. T6SS-dependent exclusion of *Aliivibrio fischeri* non-kin strains has been reported during their colonization of the light organs of the Hawaiian bobtail squid *Euprymna scolopes* (Speare et al., 2018, 2020). The ensuing symbiosis results in morphogenesis of the organs, a process that a combination of *A. fischeri* LPS and specific monomeric peptidoglycan fragments, issued from cell wall remodeling occurring during bacterial growth and considered as a sign of bacterial viability (referred to as tracheal cytotoxin; TCT), are sufficient to stimulate (Koropatnick et al., 2004). In this case, the peptidoglycan fragments are actively released during *A. fischeri* growth. In the fruit fly *Drosophila melanogaster*, recognition of peptidoglycan by the peptidoglycan recognition protein (PGRP) scavenger receptors stimulates the Immune Deficiency (IMD) pathway, similar to that of tumor necrosis factor (TNF) in mammals (Kleino and Silverman, 2014). Alternative isoforms of PGRP can determine bacterial viability: recognition of TCT activates the pathway; whereas recognition of polymeric peptidoglycan fragments (issued from bacterial killing) by a splice variant exerts an inhibitory effect of signal transduction (Neyen et al., 2016). This effectively results in a dampened immune response as reduced pathogen viability could represent a reduced threat. Such interplay also occurs in the intestinal lymphoid tissues, where the generation of IgA-producing B cells is induced following the recognition of gram-negative bacterial peptidoglycan by NOD1 in epithelial cells (Bouskra et al., 2008). Other ligands provide additional cues for microbial viability in host cytosol, such as cyclic dinucleotides sensed by the cGAS-STING and RECON pathways (Moretti and Blander, 2018; Whiteley et al., 2019); and bacterial RNA sensing by TLR8 in the endosome of mammalian epithelial cells (Ugolini et al., 2018).

Equally, it is reasonable to envision T6SS machineries and their effectors as direct inducers of immune tolerance at mucosal sites. In agreement with such possibility, host cells of the innate immune system may forge tolerance by acquiring antigens through OMVs (Shen et al., 2012; Kaparakis-Liaskos and Ferrero, 2015; Chu et al., 2016; Durant et al., 2020). The association of TseF, an iron-acquiring T6SS effector of *Pseudomonas aeruginosa*, with OMVs may represent an underappreciated role for T6SS effectors in host-microbe interplay (Lin et al., 2017). A better understanding of the activities of T6SS effectors deployed by bacterial species at the interface of mucosal surfaces will illuminate the innate immune sensing and response mechanisms to bacterial molecules released into the host milieu, during homeostasis or under stress conditions.

## T6SS Deployment by Bacterial Pathogens: Upsetting the Applecart

By its sheer density, the microbiota offers high resistance to colonization by pathogens. Indeed, pathogens are vastly outnumbered at the start of infection and must compete with the host microbiota for space and nutrients, notwithstanding the contact-dependent and -independent mechanisms of bacterial warfare. Although the T6SS was initially associated with bacterial virulence, the precise role of this apparatus in host infection has not always been clear (Hachani et al., 2016). Recently, studies have highlighted the role of the T6SS in bacterial antagonism during infection, rather than through a direct interaction with host cells. Early evidence for T6SS-mediated competition *in vivo* emerged from a transposon library screen of *Vibrio cholerae* strains for impaired colonization of the infant rabbit intestine (Fu et al., 2013). The authors found that *tsiV3*, encoding the immunity protein to the specialized peptidoglycan hydrolase effector VgrG3, is necessary to alleviate a colonization bottleneck in this model of intestinal infection. Further analysis of T6SS dynamics during *V. cholerae* colonization found that its role in commensal elimination is largely confined to the jejunum, suggesting that this antibacterial activity may be targeted toward specific microbial residents of this niche (Fu et al., 2018). The T6SS of gastrointestinal pathogens *Salmonella enterica* serovar Typhimurium and *Shigella sonnei* are also required for complete virulence, with evidence supporting a role in antagonism of members of the microbiota (Sana et al., 2016; Anderson et al., 2017). Yet, similar to *V. cholerae*, S. Typhimurium exhibited a limited target range in bacterial competition assays against gram-negative members of the microbiota, again hinting at specific targeting during infection (Sana et al., 2016). Although the abundance of proteobacterial commensals is low in comparison to members of the Bacteroidetes and Firmicutes phyla, they are enriched in many niches, for example *Acinetobacter* spp. in colonic crypts, and *Escherichia* and *Shigella* species in the sigmoid colon (Pédron et al., 2012; James et al., 2020). Due to the clash of nutritional niches between many proteobacterial gut residents and their pathogenic proteobacterial counterparts, T6SS-mediated antagonism is likely to unfold between them. Moreover, metagenomic analyses indicate the presence of species possessing T6SS^i^ components, which are absent from the Bacteroidetes subgroup, thereby supporting the notion of T6SS^i^-mediated warfare waged by commensal bacteria (Coyne and Comstock, 2019).

The induction of inflammatory host responses is a common mechanism of mass disruption by bacterial competitors, which promotes elimination of the microbial community and dysbiosis (Ackermann et al., 2008). For example, by triggering macrophage pyroptosis, an invasive subpopulation of S. Typhimurium can elicit a large inflammatory response leading to the release of pro-inflammatory cytokines from epithelial cells (Thiennimitr et al., 2012). Although this tissue-invasive S. Typhimurium subpopulation is eliminated by the subsequent infiltration of immune cells, the ensuing inflammatory response (notably the IL-22 signaling axis) reduces iron availability in the lumen. Due to its numerous metal ion acquisition systems, the luminal S. Typhimurium subpopulation is able to outcompete the commensal inhabitants and replicate in the lumen (Behnsen et al., 2014). Similarly, the secretion of cholera toxin by *V. cholerae* results in iron depletion to favor the pathogen's proliferation at the detriment of the microbiota (Rivera-Chávez and Mekalanos, 2019). The antibacterial activity of the T6SS itself can also stimulate host inflammation. Bacterial lysis mediated by the *V. cholerae* T6SS in mice mono-colonized with a commensal *E. coli* strain elicits a host transcriptional response, elevating expression of antimicrobial peptides and NF-κB signaling components (Zhao et al., 2018). NF-κB induction could be recapitulated *in vitro* using supernatants from T6SS-dependent killing assays, suggesting that MAMPs released from T6SS-mediated bacterial lysis may be the factors supporting the induction of this host response. Furthermore, El Tor pandemic strains of *V. cholerae* display higher levels of T6SS gene expression than reference clinical isolates, thus underpinning the association of T6SS antibacterial activity with pathology (Zhao et al., 2018). In the TRUC murine model for ulcerative colitis, the presence of a commensal bacterial population promotes spontaneous disease onset in this susceptible host (Garrett et al., 2007). Here, the presence of *Proteus mirabilis* and *Klebsiella pneumoniae* in this commensal community correlated with colitogenic potential (Garrett et al., 2010). Both of these species possess T6SSs that display antibacterial activity (Alteri et al., 2013; Hsieh et al., 2019), while this secretion system has also been shown to contribute to the fitness of the pathogens *in vivo* (Lery et al., 2014; Debnath et al., 2018). One can therefore contemplate a role for this secretion system in the TRUC model whereby T6SS-mediated elimination of commensal bacteria promotes an inflammatory response that cannot be restrained due to the immune genes deficiency of the host, resulting in colitis.

Non-mammalian models also support the notion of T6SS-dependent dysbiosis as a driving force for disease symptoms and pathology. A recent study found that *Pseudomonas protegens* uses antibacterial effectors to antagonize the gut microbiota of butterfly larvae, enabling tissue invasion and disease onset (Vacheron et al., 2019). Infection of *D. melanogaster* with *V. cholerae* results in diarrheal symptoms and gut inflammation (Blow et al., 2005), and the T6SS of the pathogen was found to promote mortality in a manner dependent on the presence of constituents of the microbiota (Fast et al., 2018). The IMD pathway also contributes to this pathology, suggesting that elimination of the fly gut commensal bacteria can be lethal due to exacerbated host inflammatory response (Ryu et al., 2008; Fast et al., 2018). T6SS-mediated depletion of the polymicrobial community impacts tissue repair during fly infection, mirroring the pioneering work establishing the role of the human gut microbiota in tissue homeostasis (Rakoff-Nahoum et al., 2004; Fast et al., 2020).

The competition for nutrients is a key aspect of colonization resistance in the host environment. As discussed above, microbiota niche occupancy is partly dictated by the ability to use specific carbon and nitrogen sources. Around one fifth of the genome of *Bacteroides* spp. encodes proteins involved in polysaccharide catabolism, conferring great metabolic versatility (Sonnenburg et al., 2005; Schwalm and Groisman, 2017). Besides, the host accentuates the state of nutritional immunity by sequestering metal ions upon infection to limit the replication of pathogens. Recent work by the Shen laboratory and others has revealed a role for the T6SS in nutrient acquisition, whereby the secretion of metal ion-binding proteins facilitates the uptake of zinc, iron, copper or manganese (Wang et al., 2015; Lin et al., 2017; Si et al., 2017a,b; Han et al., 2019). A *T6SS-4* mutant of *Yersinia pestis* exhibited reduced pathogenicity in an orogastric mouse model, indicating the role of this virulence factor in overcoming nutritional immunity during infection (Wang et al., 2015). It is likely that members of the microbiota utilize the T6SS for nutrient acquisition too; however, no T6SS effectors have been described to date. The role of the T6SS of bacterial pathogens in disrupting the steady state of microbiota-host ecosystems is becoming increasingly clear and underscores the importance of the microbiota in colonization resistance alongside the versatility of this secretion system.

## Direct Host Cell Contact: T6SS Encounters of the Third Kind

The T6SS versatility extends beyond its prominent antibacterial role in many gram-negative bacteria. As the most evolved member of the contractile injection systems, it delivers effectors into the extracellular milieu or directly into neighboring bacteria and/or eukaryotic targets. Many anti-eukaryotic activities of the T6SS have been described, including the manipulation of biochemical processes governing the physiology of phagocytes and epithelial cells (reviewed in Hachani et al., 2016). Furthermore, several studies have found that the T6SS can target fungal cells, and whereas the human microbiota also harbors fungi such as *Candida albicans*, these interactions within a host remain unexplored (Haapalainen et al., 2012; Trunk et al., 2018; Storey et al., 2020). A summary of T6SS effector proteins with roles distinct from direct bacterial antagonism are listed in Table 2.

**Table 2:** T6SS effectors with roles beyond bacterial antagonism.

**T6SS Effector**	**Bacterium**	**Function**	**References**
VgrG1^AD^	*Aeromonas dhakensis*	Cytoskeletal disruption	Suarez et al., 2010
TecA	*Burkholderia cenocepacia*	Inhibition of Rho GTPases	Rosales-Reyes et al., 2012; Aubert et al., 2016
TseZ	*Burkholderia thailandensis*	Acquisition of Zn^2+^	Si et al., 2017a
TseM	*B. thailandensis*	Acquisition of Mn^2+^	Si et al., 2017b
VgrG5	*Burkholderia pseudomallei*; *B. thailandensis*	Formation of multi-nucleated giant cells	Schwarz et al., 2014; Toesca et al., 2014
EvpP	*Edwardsiella piscicida*	Inhibition of inflammasome formation	Chen et al., 2017
KatN	Enterohaemorraghic *Escherichia coli*	Protection against oxidative stress	Wan et al., 2017
OpiA	*Francisella tularensis*	Phagosomal escape	Eshraghi et al., 2016; Ledvina et al., 2018
Azu	*Pseudomonas aeruginosa*	Acquisition of Cu^2+^	Han et al., 2019
TseF	*P. aeruginosa*	Acquisition of Fe^3+^	Lin et al., 2017
PldA	*P. aeruginosa*	Internalization into non-phagocytic cells	Jiang et al., 2014
PldB	*P. aeruginosa*	Internalization into non-phagocytic cells	Jiang et al., 2014
Tle4^PA^	*P. aeruginosa*	Disruption of ER homeostasis	Jiang et al., 2016
VgrG2b	*P. aeruginosa*	Cytoskeletal manipulation	Sana et al., 2012
Tfe1	*Serratia marcescens*	Membrane depolarization	Trunk et al., 2018
Tfe2	*S. marcescens*	Metabolic dysregulation	Trunk et al., 2018
VgrG1^VC^	*Vibrio cholerae*	Cytoskeletal disruption	Pukatzki et al., 2007; Ma et al., 2009
VasX	*V. cholerae*	Formation of membrane pores	Miyata et al., 2011
YezP	*Yersinia pestis*	Acquisition of Zn^2+^	Wang et al., 2015

Once pathogens gain a foothold by ousting the residing microbiota in their desired niche, they must contend with the microbial clearance mechanisms of the host. After phagocytosis by immune cells, phagosomal bacteria are subjected to the oxidative burst, where the nicotinamide adenine dinucleotide phosphate (NADPH) oxidase membrane complex produces superoxide radicals in the vacuole to destroy the engulfed microbe. During oxidative stress, such as after uptake by macrophages, enterohaemorrhagic *E. coli* (EHEC) secretes the T6SS catalase effector KatN to detoxify the local environment (Wan et al., 2017). Intriguingly, despite being important in the survival of EHEC in macrophages, the absence of KatN did not impact virulence in a streptomycin-treated mouse model. However, the T6SS itself was required for complete virulence suggesting the presence of other host cell-targeted effectors or undescribed compensatory host cell mechanisms. The T6SSs secreting metal binding effectors are upregulated upon oxidative stress, suggesting they likely play a role in defense against reactive oxygen species produced by immune cells (Wang et al., 2015; Lin et al., 2017; Si et al., 2017a,b). Indeed, the zinc-binding effector YezP of *Y. pestis* is required for intracellular survival in macrophages (Wang et al., 2015).

*Burkholderia cenocepacia*, an opportunistic pathogen of cystic fibrosis patients, resides primarily in alveolar macrophages where it resists killing (Schwab et al., 2014). The delivery of the T6SS effector TecA into the macrophage cytosol leads to the deamidation of Rho GTPases, which hampers the activity of the NADPH complex (Rosales-Reyes et al., 2012; Aubert et al., 2016). Yet, this inactivation of Rho GTPases is detected by the pyrin inflammasome, leading to caspase-1 activation, pyroptosis and inflammation (Xu et al., 2014). Inflammasomes are vital for enacting cell-autonomous immunity. Thus, they are frequently targeted by invasive bacterial pathogens (Sanchez-Garrido et al., 2020). The NLRC4 and NLRP3 inflammasomes are activated by the type III secretion system of *Edwardsiella piscicida* after phagocytosis. However, this bacterium is also able to impair the activation of caspase-1 using its T6SS effector EvpP (Chen et al., 2017). The mode of action of this effector remains elusive but appears to prevent the induction of ASC-mediated canonical inflammasome seeding by inhibition of calcium-dependent JNK activation.

The facultative intracellular pathogen *Francisella tularensis* avoids destruction by macrophages through the action of its T6SS (Nano et al., 2004). Proteomics analysis identified several T6SS effector proteins that are required for escape from the phagosome, and cytosolic replication (Eshraghi et al., 2016). One of these is the phosphatidylinositol 3-kinase (PI3K)-like effector OpiA, which remodels the phospholipid content of the phagosomal membrane to delay its maturation in the endosomal compartment, thereby facilitating pathogen escape prior to lysosomal fusion (Ledvina et al., 2018). The H2-T6SS of *Pseudomonas aeruginosa* also delivers membrane targeting effector proteins into host cells, namely the phospholipases PldA, PldB, and Tle4 (Jiang et al., 2014, 2016; Wettstadt et al., 2019). While PldA and PldB promote internalization of *P. aeruginosa* by manipulating the PI3K-Akt signaling axis, Tle4 fragments the endoplasmic reticulum, activating the unfolded protein response and autophagy. However, the benefits of these cellular modifications for the bacterium remain unclear. The T6SS yielded by bacterial pathogens targeting host cells presents a further risk to the microbiota, since the ensuing subversion of host processes affects their ecological niche. Indeed, such indirect impact has been demonstrated in the zebrafish model of cholera, where the actin-crosslinking domain of VgrG1 of *V. cholerae* stimulates peristalsis, resulting in the collapse of the resident microbial community (Logan et al., 2018). The gradual repopulation by the commensal microbiota may evict the invading pathogen despite the reversal of the numerical advantage; yet the niche must still be conducive for this repopulation to occur.

## Conclusions

The extended versatility of the T6SS enriches both the panel of virulence factors of bacterial pathogens, and the mutualism toolkit of symbiotic bacteria. The T6SS plays an underappreciated role in the maintenance of this synergistic steady state in the microbiota. Notwithstanding its original designation as a virulence factor, the T6SS is clearly beneficial to the host in facilitating stable colonization of the microbiota. Further investigation into the genetic architecture of the T6SS^iii^ of Bacteroidales, its target range, and effector-immunity repertoire will provide deeper insight into the ecology of the microbiota. Contact-dependent signaling has been described for CDI toxin delivery into immune prey (Garcia et al., 2016) and analogous processes may also be operated by T6SS effectors targeting both bacteria and eukaryotic cells. Exploring the interactions between the T6SS of commensal bacterial and host cells may illuminate the factors commandeering a homeostatic and balanced tolerogenic signaling; with broader implications in infection, diet, autoimmune and autoinflammatory disorders. In all, we describe the underappreciated roles of the T6SS at the nexus of the microbiota, host and the defense against incoming pathogens; and propose further avenues of investigation to dissect the role of this versatile secretion machine in the establishment and homeostasis of holobionts.

## Author Contributions

All authors have intellectually revised this work together and approved it for publication.

## Conflict of Interest

The authors declare that the research was conducted in the absence of any commercial or financial relationships that could be construed as a potential conflict of interest.
